# Tumours of the Skin
*An address to the British Medico-chirurgical Society, 8th December, 1965.


**Published:** 1966-04

**Authors:** D. C. Bodenham


### TUMOURS OF THE SKIN*

BY

D. C. BODENHAM

Skin tumours are so common that, directly or indirectly, they account for 48·5 per cent of all out operations in the Plastic Unit in Frenchay, and nearly half of those we deal with are malignant. So it seems appropriate that some of our experiences as a team of surgeons and pathologists in this field should be the subject of a paper.

I should first like to ask for your indulgence this evening in allowing me some licence in the interpretation of the word "tumour". The classical description of most tumours can be found in any good reference work on the subject. There is no mystery. However, when a tumour which seems clearly to belong to one particular type proves to be something different, then there is mystery and interest enough. Tumours seem to delight in mimicking their fellows, and we must be prepared, for example, to find that what appears to be a typical squamous carcinoma is in fact a non-pigmented malignant melanoma. So too, an exaggerated repair process may look and present like a malignant tumour.

I therefore chose to speak mainly about those presentations of ordinary tumour which are not usually described, but which to me at least seem to be met with as frequently as the text-book types.

The classification of my choice is not new, but has been chosen for its simplicity and directness, because I can understand it, and I offer no apology for looking back 1,800 years to Galen, who recognized three broad types of "tumour":—

1. Those according to nature (e.g. pregnancy).
2. Those exceeding nature (the repair processes).
3. Those contrary to nature (the true tumours).

### TUMOURS "EXCEEDING NATURE"

The tumours of the second group, those that are an excess of a natural process, are of particular interest and practical importance. They include the repair processes such as thickened scars and keloids, callus around fractures, and even the hump of new bone that forms on the bridge of a nose after injury. I shall deal with this group first. Consider the repair of a simple skin wound—a scratch, abrasion, or superficial burn—which causes a breach of the epithelium only, without damage to the dermis. This will heal within 7–10 days by re-growth of epithelium left undamaged in the epithelial pegs, the hair follicles, or the grease glands. The end result should be skin perfect in quality, colour and texture; there is no scar tissue and no problem.

If however healing is prevented by infection, or by damage to the deeper layers in the dermis, there must be repair of the connective tissue before epithelium can grow across the breach. The rate of healing will depend on the depth of the damage and the total area affected, and may vary from some 15 days to as much as a year or more. Normally the connective tissue begins to form as red, vascular granulation tissue; this builds up and as soon as it reaches the level of the surrounding skin, the epithelial cells from the undamaged skin edges begin to grow across the surface, at a rate of about a millimetre a day. The flatter the surface the quicker is the growth, and any islands of epithelium that have survived the injury contribute to the speed of

* An address to the British Medico-chirurgical Society, 8th December, 1965.

healing. But epithelium is not a mountaineer, nor does it like to descend into cavities or depressions, and so, if the granulations fail to fill up the hole, or if they sprout above the surface, healing is delayed or may cease.

Sometimes quite a small wound, often on a finger or on the face, is prevented from healing because it is subject to repeated damage by friction or scratching, and the granulations sprout up to form a small red, vascular tumour known as a pyogenic granuloma; this persists and closely resembles an ulcerating epithelioma or an ulcerating non-pigmented malignant melanoma. This error may not matter so much, but a mistake the other way round can be very serious. Obviously a clear history of injury to normal skin is in favour of the lesion being a granuloma, but this should never be taken for granted, and invariably the pathologist's help with the microscope must be sought. For example hypertrophic granulations on a gravitational ulcer can pose a diagnostic problem that can only be resolved histologically.

The temptation to take for histology a small piece from a tiny lesion should be resisted; it is often too small for accurate identification, and mistaken diagnosis leads to delay or to improper treatment with perhaps loss of the one opportunity for cure. The other error which is equally serious is to fail to send the pathologist anything at all. In such a case the first warning that the original lesion was malignant is the appearance of a recurrence or a metastasis. All of us have seen such tragedies, in fact everything removed should be sent to the laboratory.

The taking of a sample for histology carries with it the responsibility of ensuring that the piece is large enough and is truly representative of the lesion as a whole. In the case of small lesions this means total removal, and in larger lesions it means the taking of two or more pieces which must be adequate both in area and depth. The pathologist would add that the tissue should not be bruised or crushed by an instrument, for if it is, then the surgeon gets the answer he deserves but it is of no help to him or his patient.

After epithelium has grown across the surface and "healing" has taken place, the repair process remains active for many months. Even if this goes on quietly the scar remains red, tender and itchy. But if the activity is stepped up, the scar becomes very red and tender, hot to touch, and thicker. This state of *scar hypertrophy* may go on developing for several months, and persist for a year or more. When the build-up is extreme we call it keloid; but both scar hypertrophy and keloid are really degrees of the same condition. Gradually over a period of years the hypertrophied scar, or keloid, tends to become less vascular, paler, less itchy and tender, and it becomes softer and thinner. It is capable, however, of shrinking to cause serious contractures. Keloids may follow trivial injuries such as ear-piercing or there may be no clear history of injury at all. Scar hypertrophy, or keloid, is most prevalent amongst coloured people. It is also common in children but it is very rare in those under the age of a year. It is particularly likely to occur on the front of the chest following acne or scratches. It can be alarming to the surgeon and the patient, jeopardize an otherwise successful surgical procedure and occasionally be active enough to mimic fibrosarcoma of the skin.

Individual susceptibility is the most important factor in keloid production. Vaccination with vesication and infection produces the optimal conditions for keloid and it is not surprising that we have seen over 30 examples in the last two years. Attempts to remove these lesions surgically can lead to terrible results. For example, a vaccination vesicle of ½ cm. diameter can produce a keloid 1–2 cm. in diameter, and if this is excised, the scar will be at least 2–3 cms. long when sewn up. When this in turn becomes a keloid it will cover pehaps 25 times the original area. A good case can be made out for vaccinating all girls on that small area of buttock normally covered by a modern bathing costume. It is safe however to carry out the first vaccination on the instep because the risks of keloid in the first year of life are remote.

The process of resolution of a keloid can be accelerated by X-rays given early enough, but treatment given later is useless, and the best results follow therapy given in anticipation—before the keloid even develops. So in selected cases of known keloid tendency, we ask our colleagues in Radiotherapy to help with both pre- and post-operative irradiation. In the gross scars of burns, the symptoms of tenderness and irritation which lead to scratching and therefore provocation of the keloid may be lessened by X-rays. However, the problems of irradiating large areas and the effect on the epiphyses have to be faced. There is a theoretical case for the use of steroids, but locally they do little good and systemically the dose needs to be too high and too prolonged for safety.

Surgical treatment of keloids should be avoided whenever possible, or only undertaken after warning the patient or parents of the risks, and after taking all possible care to avoid infection and produce the best possible scar.

Since multiple active keloids may closely resemble fibro-sarcoma both to the naked eye and microscopically, it is not surprising that occasionally mistakes are made. It may seem paradoxical that a malignant condition, adequately excised, should not recur, while the benign keloid is most likely to do so. But of course the benign keloid is an excess of the natural repair process, and the specific (but unknown) factor in the individual that excites it is still present after excision.

### "CYSTS"

These lesions form a mixed group. Some are variations of a natural process and others are unnatural, some are inflammatory and some may be malignant.

The simplest and most common is the epidermoid cyst due to a blockage of a sebaceous duct. They are of the skin, and therefore fixed to it. Often they are multiple; by reason of their growth they can compress surrounding tissues to form a capsule inside which the cyst expands and from which it can be shelled out. If they become infected the inflammatory process destroys this relationship, and this cannot be done. Then if one or more parts are left behind, a complex recurrence forms and if they are to be cured this needs to be excised more as if it were malignant rather than benign.

Very occasionally spontaneous cure can occur because the overlying epithelium necroses under pressure and the cyst expels itself, or infection may itself destroy the secreting cells and the cyst shrinks.

Dermoid cysts develop beneath the skin, and being developmental faults, occur at certain sites—the majority under the outer end of the eyebrow, and under the chin behind the symphysis, or in relation to the ear (though here they usually communicate with the surface and present as a sinus). Occasionally epithelial cells are implanted by puncture wounds as on the hand to form the so-called implantation dermoid. Dermoid cysts are often much larger than they appear—their long association with underlying bone can result in marked depressions, and they have to be carefully dissected out of the bony saucer. Interesting dermoids occur in the nasal septum, discharging through the skin of the nose. Removal of these can be difficult, and is certainly not a minor operation. Dermoid cysts may occasionally calcify to form "buttons" known incorrectly as benign calcifying epithelioma.

Whenever a cyst or discharging sinus is found in the cheek, or under the chin, it is always wise to examine the teeth to look for caries or a recent filling, and to feel inside the mouth for any connection between the two. This may be in the form of a tracking sinus extending from the root pulp infection. The infection, which gets in through a point of caries, may have been sealed in by filling the cavity, having produced no more than transient toothache which was relieved when the abscess burst through the thin wall of the alveolus into soft tissue.

Cysts at the end of a finger are usually synovial in origin and contain mucus. They are particularly difficult to cure because of their complex nature.

Retention cysts of the mucous glands of the lip produce a very typical picture with a bluish translucent swelling, and at the same site regular biting of the lip and sucking can produce a similar sized lesion.

In the surgery of any of these lesions on the exposed parts, every care should be taken to achieve the best cosmetic results, by fitting the stitch line into a natural crease line. The incision is marked out, sacrificing an ellipse of skin over the cyst where it is very thin and may be adherent. The bevel of the local anaesthetic needle is then laid flat on the skin but no attempt is made to pierce the skin; pressure on the syringe piston is then asserted and kept up for some 40 seconds. It is surprising how often it is possible to get enough local anaesthetic into the skin in this way, so that when the needle is now pushed into the skin it is painless. The infiltration can then be completed. At first the incision with a knife is made shallow, to avoid cutting into the cyst, for this makes it more difficult to remove the cyst without leaving any behind. When the cyst wall is in sight fine blunt-ended scissors facilitate the rest of the operation. A fine skin hook steadies the specimen so that an assistant is not essential. Delivery is then completed and all bleeding controlled, the dead space is obliterated with a suture or two of 4/0 plain catgut, and the approximation of the edges helps in the final skin suturing. For this 5/0 or 6/0 black silk is used. Each stitch is placed 2 mm. from the skin edge and 3 mm. from its neighbour. It must be tied loosely so as not to cut in and leave stitch marks, and removed on the 6th or 7th day. If the final stitch line lies parallel to, or within a natural crease, there is a reasonable chance that the final scar will not be conspicuous.

### TUMOURS OF VESSELS

Broadly, these are of two types—tumours due to abnormalities of the blood vessels and tumours of the lymphatics. I can only speak of the blood vessels tonight.

The blood vessel tumours occur in three forms:—

### 1. *The Strawberry Naevus*

This is due, mainly, to an excess of arterioles, veins and capillaries. It appears soon after birth and grows quite rapidly for several months. The rate of growth is often alarming at first, but it slows up after a while and usually ceases by 9–12 months of age. After this the brilliant red colour deepens, becomes dusky, and then begins to fade, ultimately assuming a mottled appearance with grey patches; by the age of 7–9 years very few have persisted. Occasionally they bleed when rubbed, but this stops with light pressure, and they soon heal over with a simple protective tulle gras dressing. Treatment, whether by injection, $CO_2$ snow or irradiation should be avoided—the end-results of cure by natural processes are always best. The few that leave a surplus of tissue-paper-like skin, or a fibro-fatty mass, need tidying up by simple operation.

### 2. *The Port Wine Stain*

This is not at first a tumour, being due solely to capillary excess in the skin, which is smooth and flat. The less intense ones usually remain constant, and a few tend to fade. These can be improved by tattooing the discoloured skin with pale pigment. The deeper port wine stains tend to undergo a thickening later in life, and this gross disfigurement can only be dealt with by replacing the area with a split skin graft. When the lesions occur around the orbit, there may be an associated component on the cerebral cortex—the Sturge-Weber syndrome. Here the cortical element can produce headaches and epileptic fits.

3. *Mixed Cavernous Haemangiomata*

These are a special problem. Some of these lesions have free arterio-venous communications, and there is pulsation and a forceful bruit. The load on the heart in its attempt to maintain a blood pressure with such a wasteful by-pass can impair physical stamina, or lead to premature cardiac failure.

Rhinophyma is another condition due to an excess of a natural process. Here the tumour-like mass on the nose is due to a spongy proliferation of the sebaceous glands; though progressive, it is easily reduced surgically. Bleeding at operation can be brisk. The many sources of epithelium in the depths of the grease glands usually provide natural and quick healing within 10–12 days.

### TUMOURS "CONTRARY TO NATURE"—TRUE TUMOURS

The innocent tumours of the skin are usually known as the ordinary cellular or the pigmented naevi, the so-called birth marks, and include the papillomas; some of them are hairy, others not. They are, however, subject to infection because of the feminine habit of pulling the hairs out, and it can be very difficult to tell what is really happening when there is a chronic low-grade infection. In all such cases there is only one course, and that is to remove the whole lesion, and determine the nature of the change microscopically.

The principal reasons for removing naevi are either cosmetic, because they may cause trouble in shaving or get caught or rubbed on clothes, or the fear of malignant change. Most of us have quite a large number of them, if we include the small ones; the chances of any one becoming malignant are remote, but the slightest change in shape, colour, or size should attract immediate attention.

Any of these naevi, but particularly the pigmented ones, can become more active at puberty. This may be a natural or physiological change; fortunately true malignant changes before puberty are rare and the simple change is much more likely. Quite considerable activity in a pigmented lesion in childhood with tumourous ulceration can be alarming, particularly so because both the clinical and microscopical pictures are very difficult to distinguish from those of true malignant melanoma. This condition, known as the "juvenile melanoma of Spitz", is in fact innocent, and no recurrences have followed simple removal. Dr. Lloyd, who has made a special study of the histology of melanomas, is able to distinguish between the two, to our great relief.

After the age of 45–50 degenerative changes in the skin tend to produce the so-called seborrhoeic wart or senile acanthoma. These are rough, slightly raised patches with a biscuit brown colour, which slowly enlarge and often itch. They can be quite dark in colour and are often mistaken for a malignant condition, but they are quite innocent and it is almost safe to say they never become malignant.

A blue naevus is a blue-black, well defined, raised lesion, which occurs mostly on the face or arm. It is a compact mass of tightly packed melanocytes, and therefore a sort of melanoma. It is quite stable, and I have never seen one turn malignant.

### TUMOUR FORMATION AND BEHAVIOUR

We are slowly moving towards a better understanding of the cause of tumour formation. There is abundant evidence that a normal cell under the regular influence of stimulation from such outside sources as the sun, chemicals, or X-rays, will undergo changes. It now seems likely that viruses and hormones also play a significant part. If the stimulation is halted before a critical point is reached, then the affected cell remains dormant for many years; but if the stimuli are re-imposed, irreversible changes are initiated and the malignant process of uncontrolled growth and spread of the disease begins, and continues until halted by treatment or death.

Tumours may vary widely in their appearance and behaviour. Some invade slowly and maintain a state of balance with their blood supply; others grow fast and destroy or outgrow their blood supply, and therefore break down and ulcerate. Some produce more tissue than they destroy and grow big; others destroy more than they produce and leave craters, irrespective of the rate of growth.

Old scars from burns and X-rays may remain stable for 50 years and then become malignant. The malignant changes progress slowly to begin with, because of the poor blood supply, but once the tumour cells have penetrated to healthier tissue then the changes are greatly accelerated. Our greatest contribution to the management of skin cancer lies in the early recognition of malignant changes as shown by thickening, colour change, or ulceration, in normal skin, in pre-existing lesions or in a scar of any sort, so that early treatment can cure with minimum interference.

### BASAL CELL CARCINOMA RODENT ULCERS

The basal cells of the epidermis may proliferate to form a variety of tumours. They are quite often multiple, or one may be followed by others. In fact, rodent ulcers are so common that it is safe to say most of us will get one or more if we live long enough. The changes may be slow so that for years there is little to record; but sometimes the tumours may grow alarmingly.

The typical picture of a rodent ulcer is a pearly nodule on the face, more often near the inner corner of the eye. It has a slight depression in the centre. This may be so shallow that it can only be seen under oblique lighting. It can usually be felt as a firm ring, but in the earlier stages it needs the sensitive finger of a blind person to feel it. As it grows over a period of years, a little crust forms in the centre and may bleed. The cystic form presents as a soft, translucent, smooth, rounded swelling which may reach a size of half a cherry before it ulcerates.

Some lesions undoubtedly arise from a single focus, but others arise from more than one focus, and later coalesce. This is a most important point, because treatment must be planned to deal with the whole affected area, and it explains why recurrence is frequently seen if the whole area is not adequately treated. Here the pathologist can be of great help. The operation specimen should be oriented by the surgeon so that any residual tumour cells can be related to the post-operative area and accurately located and removed.

Pigment may be present in a rodent ulcer because of an excess of melanocytes which colour the lesion without playing any part in the malignant process. This poses a diagnostic problem. The pigmented rodent ulcer is generally well defined, and has a slightly nodular appearance not usually seen in the malignant melanoma. When a pigmented rodent ulcer ulcerates, the diagnosis can be more difficult. A clue may be given by the presence of crater; the rodent tends to destroy tissue, but a melanoma produces more than it destroys and does not leave a crater.

From time to time we see a slowly spreading lesion which remains within the epidermis, and as it spreads the central area heals over, looking almost as if there had once been a deep burn. This type, usually seen on the temple, often reaches a large size before the owner seeks advice because the progress is slow and painless, and there is no ulceration. On the trunk and limbs another form is found, which looks just like a patch of chronic eczema, or may even be mistaken for a patch of psoriasis. This is a true intra-epidermal type of rodent ulcer, and it is often multiple; as many as 20 lesions have been found on a patient.

We know how common rodent ulcer is in Australia. and even in this country the weathered faces of farmers or seamen often bear numerous lesions. A farmer came to this country from Australia to escape the sun. The damage, however, had been done, and there was not a square inch of face that did not show disease. Eventually the change to metatasizing squamous cell carcinoma occurred, the glands became involved and the disease spread. However, dark-skinned people are immune. Sandy-haired and sun-sensitive people are most susceptible, and are well advised to avoid all unnecessary exposure to bright sun, protecting their faces by hats, or oils or creams which filter out ultraviolet light.

### TREATMENT OF RODENT ULCERS

The small lesions occurring away from the eyelids and cartilage of the ears and nose will be really cured by simple excision, cauterization, or X-rays. The objective should be the same whatever method is used—namely to cure with minimum disturbance to the patient, with the least pain, quickly, and with a good cosmetic result. A piece should always be sent for histology and it is often preferable to extend the local anaesthetic a little and complete the cure by excision. Certain lesions are of a size or are so sited (for example on the eyelids, or on the cartilage of the ears or nose) that surgery is without doubt the method of choice. Simple methods of repair by local skin rearrangement, closing the defects in without causing distortion, are generally effective. The eyelid, for example, lends itself readily to wedge excision and repair. We are fortunate in Bristol in that any problem case is usually discussed amongst our colleagues and the best method adopted for that particular case.

### KERATO-ACANTHOMA, ALSO KNOWN AS MOLLUSCUM SEBACEUM

This interesting condition, which bears the same relationship to the common wart as the decorative chrysanthemum does to the humble daisy, was until recently usually diagnosed and treated as a squamous carcinoma, which it may resemble both to look at and under the microscope. Even today the picture can in every way produce a diagnostic dilemma, if there is any variation in the clinical course.

In its typical form, it starts as a sometimes tender spot, and grows to reach a sizeable lesion in a period of six weeks. At this point further growth usually ceases and a keratin core can be made out. Left alone, natural regression takes place in another six weeks, the whole process being completed in three to four months. When molluscum occurs close to the eye, or elsewhere where skin is precious, it seems wiser to remove it totally with a small margin of tissue around, and if the pathologist confirms the diagnosis, no harm is done—if not, then a wider excision is practised. The clue usually lies in a brief history of rapid growth associated with the microscopic picture of a very well differentiated squamous carcinoma under circumstances in which an undifferentiated pattern would have been expected.

### SQUAMOUS CELL CARCINOMA OR EPITHELIOMA

The middle third of the lower lip is the commonest site for epithelioma, and here any ulcer persisting for longer than two weeks should be regarded as malignant until proved otherwise. The lesion is painless, firm to feel, and bleeds easily; fortunately, it is late to metatasize. It is rarely found on the upper lip. Those which occur at the corner of the mouth, however, are more serious, spreading more rapidly and metastasizing more often. Most of the epitheliomas of lip can be cured by simple wedge excision—it is quick, positive, and gives a good cosmetic result. It is quite possible to take away over a third of the lower lip, to close the defect, and still leave freedom for dentures. If necessary the whole lip can be removed and the defect repaired in one or two stages.

### CHEMOTHERAPY

Discussion today on cancer treatment is not complete without a few words on chemotherapy. During recent years the development of toxic compounds which retard the growth of or destroy cancer cells has reached the stage when they are beginning to find a place in the routine treatment of a few selected cases.

In our experience methotrexate has given the best results in squamous carcinoma of the head and neck. The substance is fed by continuous drip into the external carotid system by a plastic catheter for a period of 2–3 weeks. Special precautions are taken to protect the bone marrow, and treatment demands a high standard of nursing care and constant vigilance, but with this the risks are small. Under favourable conditions the malignant process is reversed, and in about 10% of cases no other treatment is required. Of the remainder some 50% are benefited. The present policy is to use methotrexate primarily as a preoperative measure for major cancers of the head and neck, and in some cases of malignant melanoma of the limbs, with the purpose of facilitating surgery and improving results.

### MALIGNANT MELANOMA

The "Black Cancer," aptly so named in the past because it was only diagnosed late or even terminally, has proved to be not only far more common than ever before believed, but generally far less malignant. It affects at least 100 per year of the 3 million people in the South Western Region. In 18 years we have collected detailed case records of 514 consecutive cases treated at Frenchay, and a better understanding of the disease has emerged, particularly as to how the disease starts and how early diagnosis can be made. Two-thirds of the growths arise in a pre-existing lesion, usually a flat naevus, and the rest in apparently normal skin. Nearly three times commoner in women than in men, it is actually eleven times as common on the female leg as on the male.

Melanoma often starts in the following manner. One of a number of long-standing pigmented patches undergoes an increase in size, darkness, and colour, itches and becomes raised from the surface. Some melanomas ulcerate early and have no obvious pigment and the can be difficult to "spot". Others are typical, though it is perhaps surprising that a number have come forward only when the chiropodist found he was making no progress with his corn scraping. On the face, in the elderly, the first sign is often a brown patch or lentigo. This spreads gradually and it is fairly safe to predict that a tumour will develop.

In our series it has been observed that the average interval between first symptoms and first reporting for treatment at Frenchay is one year. A careful study has shown that patients whose tumours have been interfered with by injury, incision, curettage or incomplete excision, have a worse prognosis than those treated adequately the first time.

A property of melanoma is to destroy blood vessels, and so they bleed spontaneously either from the primary or in deposits under the skin or in lymph nodes. This accounts for sudden enlargement overnight due to distention of the deposit with blood, or bruising which appears over the deposits.

The disease progresses through stages:

*Stage* 1. The earliest stage which can be considered malignant is a carcinoma *in situ* limited to the epidermis.

*Stage* 2. The growth has progressed to invade the dermis but there is no tumour formation.

*Stage* 3. Tumour formation has occurred.

The disease spreads by direct extension, by growing along the dermal lymphatics or along the deep lymphatics. It always involves a wider area than can be seen by the naked eye. Satellite tumours are common. It is therefore understandable that limited local excision leads to early reappearance of the tumour in the scar, and a wide local excision must be the basis of treatment, the defect being made good by skin grafting.

Our series has proved big enough to reveal a number of other interesting features about the disease. For example, the peak age group for the face and head is in the 70's, with women twice as often affected as men. But lesions on the leg are commonly found in the 30–40 age group, and here there is a striking female predominance of 11:1. Fortunately, these two sites carry with them the most favourable prognosis, slightly over 70% of patients being alive and well five years later. The disease is less common elsewhere. On the trunk and sole of foot, for example, the sex incidence is nearly equal but here the prognosis is far less favourable, survival being of the order of 35% at 5 years.

It is remarkable that many of our surviving patients have had more than one operation. Several have had waves of recurrences excised and repaired by graft, and after a few years have stopped producing new lesions. By last July we had studied 20 patients who had been pregnant and it was not possible to detect any difference in the behaviour of the disease. This of course is only an interim and not a final report. As some compensation for being afflicted more often than men, women do have a slightly better prognosis, and to date, all the patients who appear to have halted their disease, after serious recurrence, have been women.

It is very significant that the 5-year survival of patients treated is as follows:—

| | |
|---|---|
| Stage 1 | 100% |
| Stage 2 | 97% |
| Stage 3 | 65% |

(If in stage 3 the lymphatics are invaded, the prognosis falls to 38%.)

The average for all our cases is 52%. If a patient survives 5 years there is an 80% of surviving another 5 years.

To summarize, the most important lessons we have learnt are:—

1. That the disease may present in an atypical form without pigment.
2. That injury or traumatic treatment worsens the prognosis.
3. That prompt and adequate first treatment by excision and graft offers the best chance of cure.
4. That one should never give up hope.

